# Supplementary material for: Protein–Protein Interactions and Quantitative Phosphoproteomic Analysis Reveal Potential Mitochondrial Substrates of Protein Phosphatase 2A-B’ζ Holoenzyme
Source: Plants (Basel). 2023 Jul 7;12(13):2586. doi: 10.3390/plants12132586 (PMC10346264; doi:10.3390/plants12132586)
Supplement: Supplementary file 1 [file plants-12-02586-s001.zip › plants-2487314-supplementary.pdf]

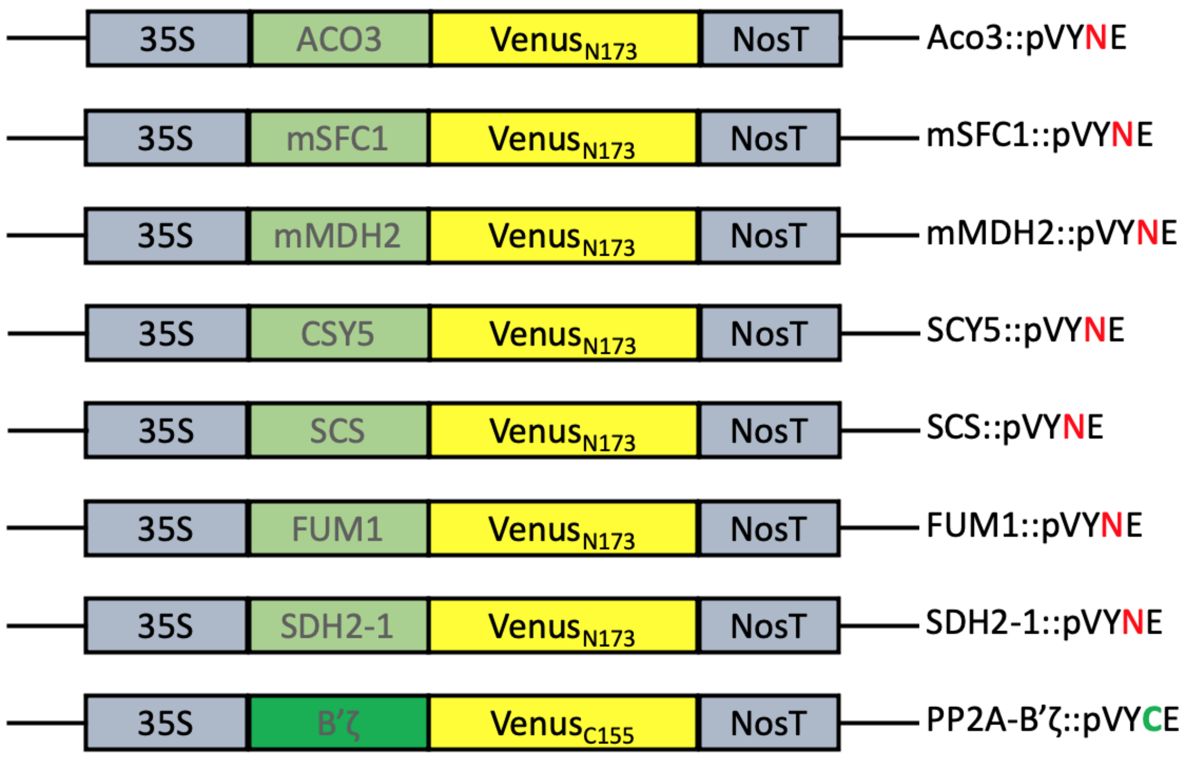

Figure S1. Schematic representation of BiFC constructs. The seven interactor candidates were fused at their respective C-terminus to the N-terminal part of Venus (pVYNE vector), while the regulatory subunit PP2A-B'ζ was fused at its C-terminus to the C-terminal part of Venus (pVYCE vector). All fusion proteins were expressed from the CaMV 35S promoter. N173: amino acids 1-173 of Venus. C155: Venus amino acids 155-238.

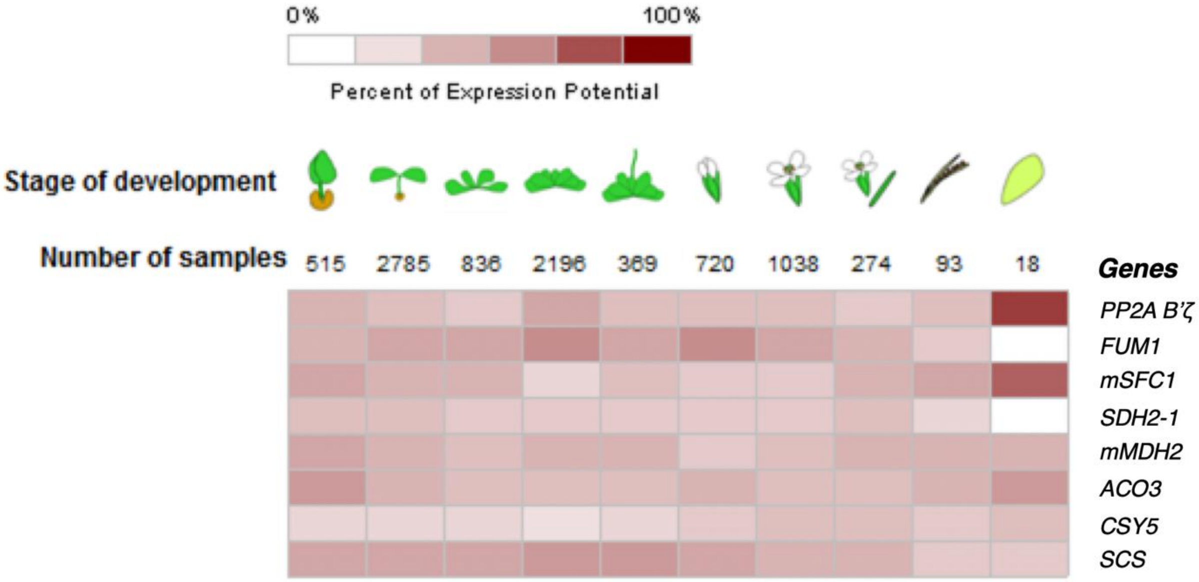

Figure S2. GeneVestigator expression study of genes coding for proteins involved in energy metabolism. All genes are expressed throughout development, and *PP2A-B'ζ*, *mSFC1* and *ACO3* are upregulated in senescent leaves.

Table S1. Oligonucleotide sequences for genotyping and cloning.

Name	Template	Sequence	Usage
Z1_150586_LP	AT3G21650	TTTTCACTTCAGAGTCAGCCG	Genotyping
Z1_150586_RP	AT3G21650	ATGGTGCATCGACCTTACATC	
Z2_107944_LP	AT3G21650	CACTCGTCGAAAAGAACTTGG	
Z2_107944_RP	AT3G21650	CCGAATCTCTTTATCGGGAAG	
Z_F	AT3G21650	ATAGTCGACATGATCAAACAGATATTTGG	Cloning (BiFC Venus)
Z_R	AT3G21650	ATGGTACCCGACCCTGTGGACTCAGA	
FUM1_F	AT2G47510	ATACTAGTATGTCGATTTACGTCGCGTCG	
FUM1_R	AT2G47510	ATATCTCGAGATCGGAGGGACCAATCAT	
mSFC1_F	AT5G01340	ATACTAGTATGGCGACGAGAACGGAA	
mSFC1_R	AT5G01340	ATATCTCGAGTAAAGGAGCATTCCGAAG	
SDH2-1_F	AT3G27380	ATACTAGTATGGCGTCTGGTTTGATCG	
SDH2-1_R	AT3G27380	ATATCTCGAGACGCTGAAGTTGCTTGAT	
mMDH2_F	AT3G15020	ATACTAGTATGTTCCGATCAATGATTGTTC	
mMDH2_R	AT3G15020	ATATCTCGAGTTGGTTGGCAAATTTGAT	
ACO3_F	AT2G05710	ATACTAGTATGTATTTAACCGCTTCATCTTCC	
ACO3_R	AT2G05710	ATATCTCGAGTTGCTTGCTCAAGTTTCT	
CSY5_F	AT3G60100	ATACTAGTATGGTGTTTTTTCGCAGCGTAT	
CSY5_R	AT3G60100	ATATCTCGAGGCGGTTCAAGCGCGTGAAGTT	
SCS_F	AT5G08300	ATACTAGTATGTCTAGACAAGTGGCAAGGC	
SCS_R	AT5G08300	ATATCTCGAGCTGCTTCAAAAGACCTCT	

Table S2. Phosphopeptides retrieved from PhosPhAt4 and the literature. Sites are experimentally verified, except if denoted as 'Predicted' on column 5.

AGI	Annotation	Phosphopeptide	Hits
AT3G14940	PPC3	^{9}MA**S**IDAQLR^{17} (*)	34
AT4G37870	PCK1	^{62}**S**AP**T**TPINQNAAAAFAAVSEEER^{84} (*)<br>^{62}**S**APTTPINQNAAAAFAAVSEEER^{84}<br>^{62}SAP**T**TPINQNAAAAFAAVSEEER^{84}<br>^{60}KR**S**AP**T**TPINQNAAAAFAAVSEEER^{84}<br>^{60}KR**S**APT**T**PINQNAAAAFAAVSEEER^{84}<br><br>^{60}KR**S**APTTPINQNAAAAFAAVSEEER^{84}<br>^{60}KRSAP**TT**PINQNAAAAFAAVSEEER^{84}	1<br>5<br>1<br>1<br>2<br><br>7<br>1
AT1G77360	PPR6	MKNV**Y**RVLK	2
AT2G05710	ACO3	^{801}DFN**S**YGSR^{808}<br>^{801}DFNSYG**S**RR^{809}<br>^{89}**T**F**S**SMASEHPFK^{100}<br>^{89}TF**S**SMASEHPFK^{100}	1<br>1<br>2<br>46
AT5G01340	mSFC1	^{94}QTAFKDSE**T**GUVSNRGRFLSGFG^{116}<br>^{235}KTRLMAQSRD**S**EGGIRY^{251}	
AT2G47510	FUM1	^{194}TLHSTLESK**S**FEFK^{207}	1
AT3G27380	SDH2-1	^{40}**SS**GGGRGSNLKTFQIYR^{56}<br>^{156}NQYKSIEPWLKRK**T**PASVPA^{175}<br>^{220}LLHANRWISD**S**RDE**YT**KERLE^{240}	
AT3G15020	mMDH2	^{174}VTTLDVVRAR**T**FYAGKSD^{191}	
AT3G60100	CSY5	^{1}MVFFRSVSAISRLR**S**RAVQQSSLSNSVRWLHSSE^{34}	
AT5G08300	SCS	^{43}A**S**DPHPPAAVFVDK^{56}<br>^{185}IGIMPG**Y**IHKPGK^{197}<br>^{296}MGHAGAIV**S**GGK^{407}<br><br><br>^{308}G**T**AQDKIK^{315}<br>^{331}IGSAM**Y**ELFQER^{342}	1<br>1<br>4<br><br><br>1<br>5

(*) Identical to phosphopeptide detected in our phosphoproteomics analysis (Table S3).

Table S3. PP2A-B'Z interactors identified by phosphoproteomics.

AGI	Annotation	Detected phosphopeptide	Probability
AT3G14940	PPC3	^{9}MA**S**IDAQLR^{17}	1
AT4G37870	PCK1	^{62}**S**AP**T**TPINQNAAAAFAAVSEEER^{84}	0.967, 0.776
AT1G77360	PPR6	^{461}**TT**QKACVLLEEMIEMGIRP**S**GVTFGR^{486}	1, 1, 0.786
